# Generic design aided robotically facade pick and place in construction site dataset

**DOI:** 10.1016/j.dib.2020.105933

**Published:** 2020-06-27

**Authors:** Ahmed Khairadeen Ali, One Jae Lee, Hayub Song

**Affiliations:** a School of Architecture and Building Science, Chung Ang University, Seoul, Republic of Korea; b Haenglim Architecture and Engineering Company, Seoul, Republic of Korea

**Keywords:** Robot in construction, Generative design, Facade design, Automation in construction, AI decision making, Robotics in architecture

## Abstract

This Dataset provides a method of optimizing robot arm, facade pick and place locations in the construction site during facade assembly activity using generative design. A set of generative algorithms are provided in the form of graphical algorithm editors. The dataset is divided into three sets, each set controlling an essential subtask of facade assembly in the construction site. the dataset is called (iFOBOT) and consist of the following sub datasets: generative tool for facade population on building envelop (iFOBOT-D), Generative algorithm aided robot spatial location optimizer (iFOBOT-B), and Quantity take-off generative (iFOBOT-L). A sample project associated with its script and outcome results are included in this dataset to guide readers how to use this tool. This dataset only focuses on robot arm and facade module placement in construction sites. This dataset can generate optimized location of robot arm workstation in jobsite while also reducing robot collision with its body and surrounding objects, 2) reducing reachability rate, 3) reducing robot time travel during operation which in result minimize risk in facade assembly and increase productivity. This dataset is in parametric format which makes it reusable with all its history data using the reproducing guide provided here. More details of how to reuse this dataset and developed tool in construction site is covered in Robot-based Facade Spatial Assembly Optimization paper [1].

Specifications table

**Table:** 

Subject	Civil and Structural Engineering
Specific subject area	Generative Design based decision making for Robotics in construction
Type of data	Figure Algorithm
How data were acquired	iFOBOT Algorithm is developed using Grasshopper (GH) graphical algorithm editor [2] inside Rhinoceros 3D [3] commercially developed software. A 3D model prototype of Royal Melbourne Institute of Technology in Melbourne, Australia (RMIT) cylindrical facade wall is designed to test this tool. Wallacei [4] generative design library is used inside GH.
Data format	Raw, analysed and filtered
Parameters for data collection	The selected building needed to have a facade module suitable for one robot arm to pick and place within the range of (100–1500) mm and weight not exceeding 100 kg. In addition, tools, libraries and robot operation language had to be compatible with GH file format for iFOBOT to work.
Description of data collection	The facade module is populated on the building elevation using iFOBOT-D which parametrically controls dimensions, scale and orientation of the facade module. KUKA 30/60 HA robot arm and facade picking location was optimized using Wallacei Generative design tool inside iFOBOT-B. The iFOBOT-L produces a workstation 3d model, graphical analysis, quantity take off and kuka krl file to run the robot arm in the construction site.
Data source location	Robot data source location: Institute: Haenglim Architecture Company City: Seoul Country: South Korea case study building: Royal Melbourne Institute of Technology in Melbourne, Australia (Latitude: −37.8047 Longitude: 144.9580)
Data accessibility	The Dataset is available in Mendeley Data Repository name: Mendeley Data Data identification number: 10.17632/vy9ygxbmzd.5 Direct URL to data: https://data.mendeley.com/datasets/vy9ygxbmzd/5
Related research article	Authors’: names:Khairadeen Ali, Ahmed; Song, Hayub; Lee, One Jae Title: Robot-based Facade Spatial Assembly Optimization Journal: Journal of Building Engineering DOI: https://doi.org/10.1016/j.jobe.2020.101556

## Value of the data

• The iFOBOT Dataset is of great value for robotically facade assembly in construction sites. it is formated in a visual algorithm where it can be reproduced and all of its historical progress visible. In addition, the Dataset or part of it can be easily integrated with other visual programming definitions since it has open ended nature. Finally, the dataset or sub dataset can be developed to cover more aspects of facade assembly without needing to remake all of the algorithm again.
• Architects, Construction Engineer, and Robot specialists can benefit from this dataset in the design and construction stage of building projects. In addition, parametric designers who are focusing on the fabrication part of design modules can benefit from the approaches and techniques provided inside this dataset to avoid fabrication problems during robotically fabrication modules. Finally, BIM specialists can use this dataset to integrate the activity of facade assembly during the construction stage with project work breakdown structure in 4D BIM and increase automation using machines in construction.
• Your third point bullet must explain how these data might be used/reused for further insights and/or development of experiments. The dataset can be used in two approaches: the first approach is using the same visual program (GH) to use or develop more sophisticated dataset. The second approach is checking how each sub dataset including iFOBOT-D, iFOBOT-B and iFOBOT-L receive and send data and what kind of hidden layers these sub datasets are using then applying the same logic in a different scripting or visual program.
• The goal behind iFOBOT dataset is to create a system that solves one specific automation problem of facade assembly using robot arms, not a final product that is applied on a single project then needed to be rebuilt for another project. This system can adapt to a variety of robot arm types and sizes; it also adapts to a variety of facade shapes, scales, and weights in different construction environments. Therefore, iFOBOT is open ended and can be enhanced to do its job more efficiently with trial and error.
• The iFOBOT dataset is not limited to the facade module but it can also be adopted by different activities and objects for pick and placing tasks by changing “pick and place orient plane” location in the algorithm.
• The Generative Algorithm in this dataset can produce a large number of alternative facade assembly options and rank them according to objectives defined in

## 1 Data description

**Fig. 1:**
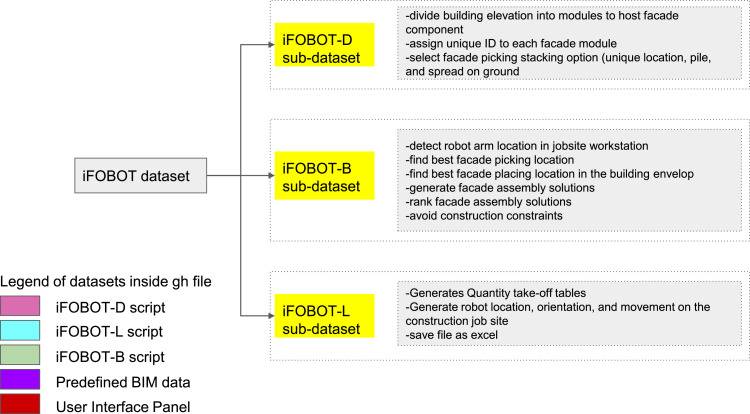
iFOBOT dataset including sub-datasets (iFOBOT-D, iFOBOT-B, and iFOBOT-L); legend of datasets inside grasshopper file color coded.

The Dataset is designed to optimize façade pick and place activity in the construction jobsite using robot arm. This dataset contains visual algorithm script and 3D model of construction jobsite. The dataset repository can be found in Mendeley data as mentioned in Data accessibility section. It was built using grasshopper graphical algorithm editor. Fig. 1 divides iFOBOT in practice into 3 categories: 1) A user interface was built with sliders, dropdown lists, and action buttons to start activating iFOBOT sub algorithms and control the input parameters of the system, 2) 3D model visual simulation of Robot arm, facade stacking option, facade placing workstation and collision objects surrounding this activity in construction site, 3) output spreadsheets of workstation details including GIS locations, quantities, type, and dimensions needed to implement iFOBOT virtual simulation in physical reality and Kuka krl script needed to run the robot arm in physical reality. the iFOBOT-B sub-dataset include detail algorithm structure the generative tool Wallacei [4] used in iFOBOT-B to achieve three fitness objectives: 1) Collision- where this sub dataset calculates robot collision rate and 0 indicate there is no collision and more than zero indicate there is a collision between robot and its body or surrounding objects. As a result iFOBOT-B exclude any simulation iteration that has collision value of more than 0. 2) Reachability- this objective calculates if the robot can manifest the pick and place process without having to extend its body to out of reach positions. 3) Time value- this objective calculates robot arm time travel and tries to minimize it in order to increase productivity and reduce robot arm operation time.

**Fig. 2:**
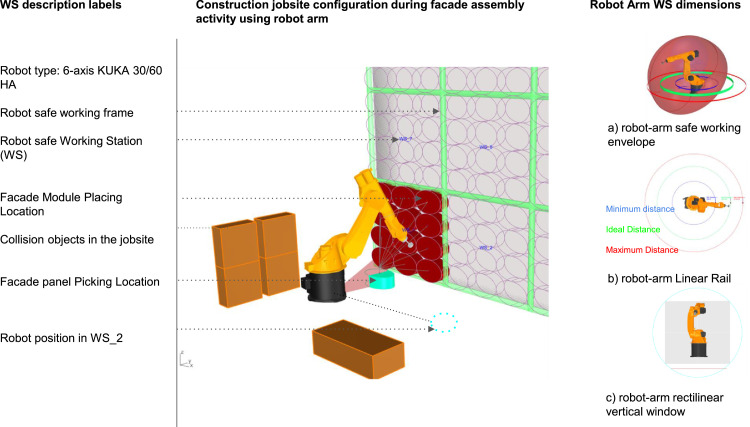
Diagram illustrating the design of the experiment for the simulation of the robotic fabrication Working Station (WS) segmentation, positioning, reachability, collision parameters.

The iFOBOT-B sub-dataset also includes the relationship logic built behind iFOBOT-B to get the desired objective in an efficient method. iFOBOT-B calculate and generate safe working frame by analysing robot arm positioning, reachability, joints, collision and travel distance as depicted in Fig. 2 (component a). Furthermore, iFOBOT-B calculate minimum distance and maximum distance a robot arm can reach and try to do most of the pick and place work while in the ideal distance as shown in Fig. 2 (component b). Finally, IFOBOT-B generated vertical window calculation from the robot arm safe envelope specifications and simulation of the robot positioning, segmentation, collision parameters, and reachability. This working window is used to divide building wall into Working Stations (WS) as shown in Fig. 2 (component c). This sub-dataset illustrates the relationship triangle between robot arm (point A), Picking location (Point B) and Placing location (point C). The generative algorithm outputs many iterations and has their rankings associated with the generation and individual information. Artificial Intelligence (AI) built in iFOBOT-B helps designers in decision making of which robot simulation is best fitting in the construction site. the raw dataset in the repository consist of five files: 1) 3D model Rhinoceros file (.3dm) of sample building that is integrated with the GH file, 2) Grasshopper definition of iFOBOT that include all sub datasets in one file (.gh), 3) BIM model platform in Revit WIP (.rvt), 4) an excel sheet sample output of iFOBOT-L (.xlsx), 5) a video describing the visual algorithm dataset of iFOBOT (iFOBOT_VIDEO.mp4), 6) screenshots of Rhinoceros and Grasshopper files to increase readability, and 7) Readme file that contain brief instructions on how to use the dataset (.txt) as illustrated in Fig. 1., component iFOBOT-L sub-dataset.

## 2 Experimental design, materials and methods

The iFOBOT dataset includes a tool for optimizing robot arm, facade pick and placing location using generative design inside a parametric algorithm editor grasshopper environment. This dataset initial concept is inspired by the work of D. Garber of facade assembly using robot arm [5]. A developed dataset uses an open ending approach which means it can be back propagated to its initial inputs and changing these inputs will change the results also. All the dataset is self-dependent inside GH to reduce compatibility issues between software [6]. The iFOBOT dataset contains three sub datasets inside one grasshopper environment file. First sub dataset, iFOBOT-D that controls the facade subdivision on the building elevation using lunchboxe [7] library inside GH. After that it extracts facade picking and placing point planes and direction needed and feed it to the robot arm gripper for assembly purposes. iFOBOT-D dataset also contain facade stacking options (unique place, side by side, and stacked) and can be extended to have more facade stacking options in the construction picking activity. The iFOBOT-D also sends all x,y,z coordinates of facade picking and placing locations to iFOBOT-L for quantity take off purposes.

Second subdataset, iFOBOT-B contains an algorithm to control Kuka Prc robot arm movement, robot arm safe working window calculator, and gripper tool movement, and generative algorithm for optimization data [8]. In order to run iFOBOT-B using the kuka robot arm, an educational licence needs to be acquired from robotics in architecture [9]. To reproduce the same results in the repository, open Rhinoceros WIP .3dm inside the revit environment then initiate grasshopper and open .gh file provided inside the repository. The pick and place process logic is as following: Point to Point movement (PTP) start position to picking offset position, Activate vacuum tool, Approach and catch the facade module, Move to picking offset position, Linear movement move to placing offset position, Move to placing position, Deactivate vacuum tool, PTP move to end position. The construction jobsite 3D model is visualized in the Rhinoceros file. It contains building wall, façade module, robot arm, working station and collision objects that might exist in the construction site while doing façade assembly activity as depicted in Fig. 2. The Façade Assembly activity in the construction jobsite needs open space for robot arm location, façade picking location and travel space for the robot arm to pick a façade module and place it on the wall. Therefore robot arm safe working envelope -see Fig. 2, component a- needs to be considered if there are any other activities in the same location and time. In Addition the collision objects in the 3d model of jobsite represent any restriction area or object that the robot arm should avoid while doing façade pick and place activity.

Third dataset, iFOBOT-L contains algorithms that generate spreadsheet quantity take-off that can be used in the construction stage for physical facade assembly guidance. In addition, it generates a kuka krl language file that is used to run the robot arm. Finally, it connects BIM environment to iFOBOT for data integration with schedule and building model. iFOBOT dataset is subcategorized into small packages of algorithms. Each package is color coded as shown in the legend of Fig. 1. The packages contain iFOBOT-D script, iFOBOT-L script, iFOBOT-B script, predefined BIM data, and User Interface Panel. The Grasshopper file title iFOBOT.gh contains all mentioned packages with colour code assigned clearly. Authors also created a video titled iFOBOT that shows all packages inside grasshopper files to increase readability and aid other researchers reproduce targeted results.

## Declaration of Competing Interest

The authors declare that they have no known competing financial interests or personal relationships which have, or could be perceived to have, influenced the work reported in this article.

## Appendix Supplementary materials

Image, application 1
